# Enteral Nutrition Safety and Outcomes of Patients with COVID-19 on Continuous Infusion of Neuromuscular Blockers: A Retrospective Study

**DOI:** 10.1155/2023/8566204

**Published:** 2023-06-28

**Authors:** Hasan M. Al-Dorzi, Reem Yaqoub, Reema Alalmaee, Ghafran Almutairi, Allulu Almousa, Leen Aldawsari

**Affiliations:** ^1^College of Medicine, King Saud Bin Abdulaziz University for Health Sciences, King Abdullah International Medical Research Center and Intensive Care Department, King Abdulaziz Medical City, Ministry of National Guard Health Affairs, Riyadh, Saudi Arabia; ^2^College of Medicine, King Saud bin Abdulaziz University for Health Sciences, Riyadh, Saudi Arabia

## Abstract

**Background:**

Intravenous infusions of neuromuscular blocking agents (NMBAs) and prone positioning are recommended for acute respiratory distress syndrome (ARDS) due to COVID-19. The safety of enteral nutrition (EN) during these treatments is unclear. This study assessed EN tolerance and safety during NMBA infusion in proned and nonproned patients with ARDS due to COVID-19.

**Methods:**

This retrospective study evaluated patients who were admitted to a tertiary-care ICU between March and December 2020, had ARDS due to COVID-19, and received NMBA infusion. We assessed their EN data, gastrointestinal events, and clinical outcomes. The primary outcome was gastrointestinal intolerance, defined as a gastric residual volume (GRV) ≥500 ml or 200–500 ml with vomiting. We compared proned and nonproned patients.

**Results:**

We studied 181 patients (mean age 61.2 ± 13.7 years, males 71.1%, and median body mass index 31.4 kg/m^2^). Most (63.5%) patients were proned, and 94.3% received EN in the first 48 hours of NMBA infusion at a median dose <10 kcal/kg/day. GRV was mostly below 100 ml. Gastrointestinal intolerance occurred in 6.1% of patients during NMBA infusion and 10.5% after NMBA discontinuation (similar rates in proned and nonproned patients). Patients who had gastrointestinal intolerance during NMBA infusion had a higher hospital mortality (90.9% versus 60.0%; *p*=0.05) and longer mechanical ventilation duration and ICU and hospital stays compared with those who did not.

**Conclusion:**

In COVID-19 patients on NMBA infusion for ARDS, EN was provided early at low doses for most patients, and gastrointestinal intolerance was uncommon in proned and nonproned patients, occurred at a higher rate after discontinuing NMBAs and was associated with worse outcomes. Our study suggests that EN was tolerated and safe in this patient population.

## 1. Introduction

COVID-19 is a disease caused by a novel coronavirus strain, the severe acute respiratory syndrome coronavirus 2 (SARS-CoV-2). The virus causes complications in various systems; however, it predominantly attacks the respiratory system and may manifest as acute pneumonia leading to acute hypoxemic respiratory failure, acute respiratory distress syndrome (ARDS), and dysfunction of other organs [1–4]. Patients often require admission to the intensive care unit (ICU) and invasive mechanical ventilation [1–4]. Such patients are often treated early after intubation with an intravenous infusion of neuromuscular blocking agents (NMBAs), usually for 1–3 days [5–8], with or without prone positioning [7–10]. NMBA infusion for patients with ARDS facilitates oxygenation and reduces ventilator dys-synchrony, ventilator-induced lung injury, and barotrauma [8, 11]. Prone positioning promotes homogeneous aeration of the lung, decreases shunting, and reduces ventilator-induced lung injury [12].

Prolonged fasting has been associated with prolonged ICU stays, increased infectious complications, and higher mortality rate [13]. Hence, early enteral nutrition (EN), within 24–48 hours of ICU admission, has been recommended [14, 15], may prevent malnutrition, preserve muscle mass, and reduce mortality [16, 17]. Adequate EN is frequently hampered by gastrointestinal intolerance that includes vomiting, diarrhea, abdominal distension, constipation, and increased gastric residual volumes (GRVs) [18]. There are also safety concerns about EN during NMBA infusion and prone positioning [19, 20]. NMBA infusion, which is often accompanied by the use of intravenous sedatives and vasopressors, may increase the risk of vomiting and ileus and hence gastrointestinal intolerance [19]. Prone positioning may also increase the risk of regurgitation and aspiration [19, 20].

The ideal approach when treating patients with COVID-19 receiving intravenous NMBA infusion while on mechanical ventilation is the concomitant use of EN. Whereas the Society of Critical Medicine made no recommendation regarding nutritional requirements specific to patients receiving NMBA infusion [21], the European Society of Intensive Care Medicine suggested that EN should not be delayed merely because of NMBA infusion [22]. Due to the lack of quality evidence, the safety and tolerance of EN during NMBA infusion with and without prone positioning need further study [19]. We aimed to evaluate whether EN was tolerated and safe during NMBA infusion for critical COVID-19 with acute hypoxemic respiratory failure/ARDS.

## 2. Materials and Methods

The STROBE (STrengthening the Reporting of OBservational studies in Epidemiology) guidelines [23] were followed in reporting this study.

### 2.1. Study Design, Setting, and Participants

The study was a retrospective study that was approved by the Institutional Review Board of the Ministry of National Guard Health Affairs and was performed in accordance with its ethical standards. The study was conducted in the adult ICUs of King Abdulaziz Medical City in Riyadh, Saudi Arabia. The hospital was a tertiary-care center with >1000 beds. During the first wave of the COVID-19 pandemic in the spring of 2020, four ICUs (ICU-A with 28 beds, ICU-B with 10 beds, ICU-C with 16 beds, and ICU-D with 16 beds) were designated to care for patients with severe COVID-19 [24]. These units functioned as closed ICUs with board-certified ICU consultants leading the provision of medical care 24 hours/7 days a week [24]. We included all consecutive patients with severe COVID-19, confirmed by reverse transcription polymerase chain reaction, who were admitted to the ICUs between March 1, 2020, and December 31, 2020, received invasive mechanical ventilation, and had continuous NMBA infusion (cisatracurium besylate) for at least 6 hours as part of the management of moderate-severe ARDS. We excluded patients who did not receive any EN during the NMBA infusion.

In this study, treatments with NMBA infusion and/or prone positioning in our ICU were at the discretion of the treating ICU team. Patients with ARDS and persistent hypoxemia (ratio of arterial oxygen partial pressure to fractional inspired oxygen <150) after intubation were usually given NMBA infusion (cisatracurium besylate infusion at 0.5–3 mcg/kg/min with dose titrated to achieve a train-of-four of 2/4 on peripheral nerve stimulation) in addition to intravenous sedative (propofol and/or midazolam) and narcotic (fentanyl) infusion. If hypoxemia persisted, prone positioning was performed, according to an ICU protocol. The head of the patient was kept elevated at 20–30 degrees in the prone position. The treating ICU team decided on starting EN, including the initial hourly volume. The ICU dietician selected the EN formula and determined the caloric goal and hourly EN volume, usually after discussion with the ICU team, especially for proned patients. The ICU nurses used an evidence-based EN protocol [15, 25] and routinely checked GRV every 4 hours by aspirating the feeding tube using a syringe and recorded vomiting episodes and bowel movements.

### 2.2. Data Collection

The collected data included demographics, comorbid conditions, date of intubation, date of initiation and discontinuation of NMBA infusion, date of initiation of EN, EN formula (energy-dense versus regular formula), total volume of delivered EN per day for up to the first 7 days, and use of parenteral nutrition. We defined early EN as EN that started within the first 24 to 48 hours of NMBA infusion. We calculated the hourly and daily caloric intake based on the delivered volume and the EN formula.

To assess EN safety and tolerance, we also collected data on frank aspiration incidents, diagnosis of ICU-acquired pneumonia based on the presence of a bacterial culture from deep tracheal aspirate with clinical features pneumonia (new infiltrates on chest X-ray) during ICU stay, vomiting episodes, bowel movements, and GRVs (per check and per 24 hours) during NMBA infusion and in the 48 hours after stopping NMBAs and use of prokinetics (metoclopramide and erythromycin) for gastrointestinal intolerance. In this study, the primary outcome was gastrointestinal intolerance. As there is no agreement on its definition and GRV is frequently considered its surrogate [18], we defined gastrointestinal intolerance as having a GRV ≥500 ml on a single check or 200–500 ml with vomiting [15, 18]. Diarrhea was defined as having ≥3 loose or liquid bowel movements per day [26]. We also noted tracheostomy events, duration of mechanical ventilation, length of stay in the ICU and hospital, and vital status at ICU and hospital discharge (secondary outcomes).

### 2.3. Statistical Analysis

The study patients were categorized into two groups depending on whether they received prone positioning. The results were presented as frequency and percentage for categorical data and mean and standard deviation or median and interquartile range (IQR) for continuous data. The Chi square or Fisher's exact test was used to compare categorical variables and Student's *t*-test or Mann–Whitney *U* test to compare continuous variables. As GRV might be proportional to the delivered EN volume, we correlated GVR per day with the delivered EN volume per day using the Pearson correlation and reported Pearson's r. We used Statistical Package for the Social Sciences version 21 (SPSS) for all statistical analyses. All statistical tests were considered significant at *p* value <0.05.

## 3. Results

### 3.1. Characteristics of Patients

During the 10-month study period (March to December 2020), 476 patients were admitted to the ICUs with confirmed SARS-CoV-2 infections. Among these patients, 181 (38.0%) received continuous NMBA infusions for the management of acute hypoxemic respiratory failure/ARDS due to COVID-19. NMBA infusion was for only 6–23 hours in 4 patients, 24–48 hours in 16 patients, 49–72 hours in 38 patients, and >72 hours in 123 patients. Their mean age was 61.22 ± 13.66 years, and most (71.1%) were males with a median body mass index of 31.4 kg/m^2^ (IQR: 26.7, 36.8). Most (115/181; 63.5%) patients had prone positioning while on NMBA infusion. The characteristics of the study patients are described in Table 1. The baseline characteristics of proned and nonproned patients were similar, except for proned patients having a lower body mass index and white blood cell count and a higher hemoglobin level. Most (76.8%) patients received systemic corticosteroids as part of COVID-19 management without significant differences between proned and nonproned patients (*p*=0.33).

### 3.2. Enteral Nutrition Data

Before using NMBA infusions, most (112/181; 61.9%) patients did not receive EN. EN was provided for 123/181 (67.9%) patients on the first day, 167/177 (94.3%) on the second day, 153/161 (95.0%) on the third day, and 123/123 (100%) on the fourth day of NMBA infusion. Among patients receiving EN, energy-dense formulas (1.5–2.0 kcal/ml) were used in 24/69 (34.8%) patients on the day before NMBA infusion, 44/123 (35.8%) on day 1, 66/167 (39.5%) on day 2, 61/153 (39.9%) on day 3 of NMBA infusion, and 55/123 (44.7%) on day 4 of NMBA infusion. There was no significant difference in the use of energy-dense EN formulas between proned and nonproned patients.

**Figure 1:**
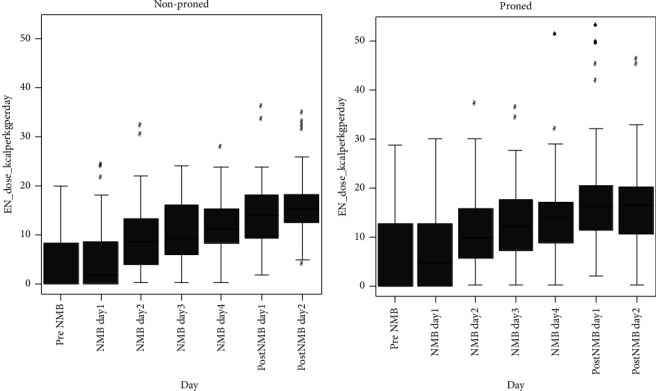
Boxplots of the caloric intake in proned and nonproned patients with COVID-19 on intravenous infusion of neuromuscular blockers for acute respiratory distress syndrome. The boxplots show the 25th, 50th, and 75th percentiles (box), 10th and 90th percentiles (whiskers), and outliers.

The caloric intake was lowest on the first day and gradually increased but remained moderate for both proned and nonproned patients (Table 2 and Figure 1). For nonproned patients, the median caloric intake per kg per day was 0 (IQR: 0–9.0) on the day before NMBA infusion, 1.7 (IQR: 0–8.6) on day 1 of NMBA infusion, 8.6 (IQR: 3.9–13.4) on day 2, 9.5 (IQR: 5.8–16.0) on day 3, 11.2 (IQR: 8.2–15.3) on day 4, 14.0 (IQR: 9.1–18.5) on day 1 postinfusion and 15.2 (IQR: 12.3–18.3) on postday 2 (Figure 1). For proned patients, the median caloric intake per kg per day was 0 (IQR: 0–13.2) on the day before NMBA infusion, 4.6 (IQR: 0–12.8) on day 1 of NMBA infusion, 9.8 (IQR: 5.7–16.0) on day 2, 12.1 (IQR: 7.2–17.7) on day 3, 14.1 (IQR: 8.7–17.5) on day 4, 16.2 (IQR: 11.3–20.5) on day 1 postinfusion, and 16.7 (IQR: 10.5–20.3) on postday 2 (Figure 1). There were no significant differences in the caloric intake between proned and nonproned patients on any of these days. None of the patients received parenteral nutrition.

### 3.3. Gastrointestinal Intolerance

GRVs were mostly below 100 ml in most patients. The median GRV was 23 ml (IQR: 0, 150) before NMBAs, 10 ml (IQR: 0, 80) on day 1, 60 ml (IQR: 10, 150) on day 2, 70 ml (IQR: 20, 180) on day 3, and 90 ml (IQR: 30, 180) on day 4 of NMBA infusion.

**Figure 2:**
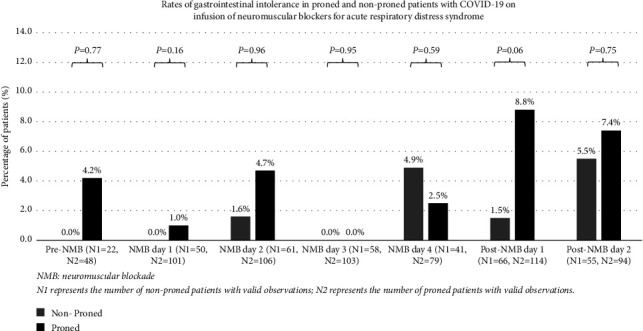
Gastrointestinal intolerance rates of patients who received continuous infusion of neuromuscular blockers. *p* values between the proned and nonproned groups were >0.1 on all days except post-NMB day 1 (*p*=0.06).

Gastrointestinal intolerance while receiving NMBA infusion occurred in only 11/181 (6.1%, 95% confidence interval: 3.1–10.6%) patients: 2.9% of patients on the day before, 0.7% on day 1, 3.6% on day 2, 0% on day 3, and 3.3% on day 4 of NMBAs (Figure 2). There were no significant differences in the rates of gastrointestinal intolerance between proned and nonproned patients on these days. Gastrointestinal intolerance was more common after discontinuing NMBA and occurred in 19/181 (10.5%) patients: 6.1% of patients on day 1 and 6.7% on day 2 after discontinuation of NMBAs (Figure 2). Patients who were proned had higher rates of gastrointestinal intolerance on day 1 post-NMB(*p*=0.06). The correlation between total GRV per day and EN volume per day was significant only on the first day (*r* = 0.435, *p* < 0.001) and fourth day (*r* = 0.472, *p* < 0.001), but not on the second and third days.

Vomiting episodes were rare during NMBA infusion, occurring in 7 patients (3 proned patients and 3 unproned patients on day 3 and 1 proned patient on day 4). Metoclopramide was used in 68 (37.6%) patients: 33.0% of proned and 46.2% of nonproned patients (*p* = 0.08). Only one patient received the combination of metoclopramide and erythromycin as prokinetic agents. None of the patients in the study had a documented aspiration event. Diarrhea was more common, occurring in 3.9–10.5% of patients per day during NMBA infusion (Table 2).

### 3.4. Other Outcomes

The other outcomes are shown in Table 3. Out of the 181 patients, 58 (32.0%) developed ICU-acquired pneumonia with no significant difference among proned and nonproned patients. ICU-acquired pneumonia occurred in 5/11 (45.5%) patients who had any gastrointestinal intolerance while on NMBA infusion compared with 53/170 (31.2%) patients who did not have any gastrointestinal intolerance (*p*=0.33). Only 35 (19.4%) of the patients underwent tracheostomy with no difference between proned and nonproned patients. Also, the tracheostomy rate was similar between those who had any gastrointestinal intolerance and those who did not while on NMBA infusion (18.2% versus 20.0%, *p*=1.0). As for the duration of mechanical ventilation and ICU and hospital lengths of stay, they were similar in proned and nonproned patients (Table 3). On the other hand, patients who had gastrointestinal intolerance while on NMBA infusion had a longer duration of mechanical ventilation (median of 27.0 days (IQR: 19.0–32.0) versus 12.0 days (IQR: 8.0–18.0, *p* < 0.001), and a longer stay in the ICU (median of 29.0 days (IQR: 17.0–38.0) versus 15.0 days (IQR: 10.0–22.3, *p*=0.004) and hospital (median of 31.0 days (IQR: 20.0–41.0) versus 22.0 days (IQR: 14.0–33.0, *p*=0.051) compared with those without intolerance.

The ICU and hospital mortality rates were similar in proned and nonproned patients (Table 3) and in patients who had early EN within 24 hours (ICU mortality: 72/122 (59.0%) versus 33/59 (55.9%), *p*=0.69; hospital mortality: 75/122 (61.5%) versus 37/59 (62.7%), *p*=0.87) or within 48 hours (ICU mortality: 96/169 (56.8%) versus 9/12 (75.0%), *p*=0.22; hospital mortality: 103/169 (60.9%) versus 9/12 (75.0%), *p*=0.54) compared with delayed EN. However, hospital mortality was higher for patients who had any gastrointestinal intolerance (10/11 patients (90.9%) versus 102/170 (60.0%) patients who did not have any gastrointestinal intolerance, *p*=0.05).

## 4. Discussion

In this study, we evaluated the safety and tolerance of EN during NMBA infusion with and without prone positioning in moderate-severe ARDS due to COVID-19. We found that EN was provided early at low doses for most patients; gastrointestinal intolerance was uncommon during NMBA infusion in proned and nonproned patients; gastrointestinal intolerance occurred at a higher rate after discontinuing NMBAs; and gastrointestinal intolerance was associated with a worse outcome.

In the current study, early EN was provided at low doses (<10 kcal/kg/day during NMBA infusion and around 15 kcal/kg/day after NMBA discontinuation) for most patients while on NMBA infusion with and without prone positioning and with frequent use of energy-dense EN formulas. Using propensity score matching, a retrospective study showed that early EN in patients receiving NMBA infusion was safe [27]. Early EN has been recommended in critical COVID-19 patients at trophic or hypocaloric doses with advancing EN dose slowly as tolerated over the first week of critical illness to meet the energy goal of 15–20 kcal/kg actual body weight [28]. This recommendation included proned patients [28]. The EN dose in our study was in line with the clinical practice guidelines [28]. However, a multicenter study from Singapore observed that the minimum caloric of 15 kcal/kg was achieved in only 39/74 (54%) patients with critical COVID-19 [29]. Energy-dense EN formulas have been suggested to safely deliver more calories, especially during prone positioning [19]. However, recent data suggested that energy-dense feeding may increase GRV and delay gastric emptying [30].

In our study, gastrointestinal intolerance was uncommon during NMBA infusion occurring in only 6.1% of patients. There was also no recorded aspirational event. Gastrointestinal intolerance is common in critically ill patients, with a systematic review of 72 studies estimating its prevalence at 38% (95% confidence interval: 31–46%) [31]. In a retrospective study of 52 critically ill patients with COVID-19 not receiving NMBA, gastrointestinal intolerance occurred in 18 patients (32.4%) within the first 7 days [32]. Whether NMBA infusion increases the risk of gastrointestinal intolerance in ARDS is unclear. Gastrointestinal intolerance was not studied in the randomized controlled trials that evaluated NMBA infusion in ARDS [33, 34]. One prospective study found similar GRVs in 20 patients while receiving opioid infusion versus opioid and cisatracurium infusion (95 ± 76 ml and 105 ± 90 ml two hours after 200 ml of tube feeding, respectively) [35]. Another study found that 10/47 patients (21%) admitted to a trauma ICU had gastrointestinal intolerance to EN during NMBA infusion for ≥48 hours [36]. Besides being tolerated, our findings suggested that EN during NMBA infusion was safe. The clinical practice guidelines of the European Society of Intensive Care Medicine suggested that EN should not be delayed merely because of NMBA infusion [22]. Whether prone positioning, which is a common concomitant treatment for patients with ARDS, increases the risk of gastrointestinal intolerance is also unclear. We found no difference in gastrointestinal intolerance between proned and nonproned patients receiving NMBA infusion. A systematic review of studies that assessed early EN during prone positioning in patients receiving invasive mechanical ventilation found that five out of six studies reported no differences in GRV between supine and prone positions [37]. One study reported a higher rate of the need to stop EN while in the prone position [38], while another did not [37, 39]. Vomiting episodes were more common in the prone position when EN was administered over 18 hours rather than 24 hours [37, 40]. The rates of ventilator-associated pneumonia, lengths of stay, and mortality were similar between supine and prone positions [37]. One prospective comparative study showed lower mortality in patients receiving a 24-hour versus 18-hour administration protocol [37, 40]. Additional studies were recently reported on COVID-19 patients. In a retrospective study in patients with ARDS due to COVID-19, gastrointestinal intolerance was observed in 30.8% of 57 patients in the prone position and 23.2% of 69 patients in the supine position (*p* = 0.81) [41]. In another multicenter retrospective study conducted in 83 critically ill patients with COVID-19, the prone position was not associated with a higher rate of high GRV (≥250 mL) [29]. Our study and the other studies indicate EN is well tolerated in patients with ARDS while in the prone position. The lower rate of gastrointestinal intolerance in our study compared to other studies could be related to the duration of assessment (up to 4 days on NMBA infusion) and the moderate hourly EN volume, which may correlate with GRV. Additionally, head-of-bed tilting to 20–30 degrees, administering EN over 24-hour rather than shorter durations, employing an EN protocol, using prokinetics, and close monitoring may have contributed to the low rate of intolerance in our study and are recommended when providing EN while in the prone position [19, 28]. Whether postpyloric EN during the prone position and intravenous NMBA infusion would reduce gastrointestinal intolerance is unclear and needs further studies [37].

In our study, neither early versus late EN nor prone versus supine position was associated with mortality. A meta-analysis of four studies in critically ill patients with COVID-19 found that early EN was significantly associated with lower mortality risk (risk ratio: 0.89, 95% confidence interval: 0.79–1.00, *p*=0.05) [42]. Prone positioning reduces mortality in moderate-severe ARDS in non-COVID-19 patients [43], but its effectiveness in intubated patients with COVID-19 is not clear [44]. In the current study, gastrointestinal intolerance was associated with worse outcomes (longer duration of mechanical ventilation, longer stay in the ICU and hospital, and higher mortality). There was no documented aspiration event associated with gastrointestinal intolerance events to explain worse outcomes. Unwitnessed or microscopic aspiration may have occurred. Besides, gastrointestinal intolerance may be a manifestation of acute gastrointestinal injury, which is a manifestation of multiorgan dysfunction [45].

The study's findings should be interpreted taking into consideration its strengths and limitations. The sample size is larger than that of most published studies on this topic, and we collected detailed data on EN and gastrointestinal intolerance. On the other hand, our study is a single-centered retrospective study, which limits the generalizability of our findings and questions causality in the reported associations. Moreover, our findings may be the result of unmeasured confounders. The low rate of gastrointestinal intolerance prevented us from performing a multivariable regression analysis to evaluate its predictors. We also did not obtain additional data on protein intake and whether the feeding tube was intragastric or postpyloric.

## 5. Conclusions

In this study of COVID-19 patients on NMBA infusion for ARDS, EN was provided early at low doses for most patients, gastrointestinal intolerance was uncommon in proned and nonproned patients, occurred at a higher rate after discontinuing NMBAs, and was associated with worse outcome. Our study suggests that EN given at low doses with the use of a 24-hour administration protocol was tolerated and safe in this patient population. These findings were similar in proned and nonproned patients. The optimal dose and volume of EN in patients treated with NMBA infusion and/or prone positioning for ARDS need further evaluation.

## Figures and Tables

**Table 1:** Baseline characteristics of the study patients.

	Nonproned *N* = 66	Proned *N* = 115	*p* value
Age (yrs), mean ± SD	62.0 ± 12.8	60.9 ± 14.1	0.62
Male gender, *N* (%)	51 (78.46%)	77 (66.97%)	0.10
BMI (kg/m^2^), median (Q1, Q3)	32.8 (28.8, 39.8)	30.2 (25.7, 36.3)	0.01

*Comorbidities, N (%)*			
Diabetes	49 (74.2%)	80 (69.6%)	0.50
Hypertension	44 (66.7%)	74 (64.3%)	0.75
Stroke	4 (6.1%)	6 (5.2%)	1.0
Liver disease	1 (1.5%)	1 (0.9%)	1.0
Asthma	5 (7.6%)	8 (7%)	1.0
Cancer	5 (7.6%)	4 (3.5%)	0.29
Heart failure	7 (10.6%)	8 (7%)	0.39
Chronic kidney disease	10 (15.2%)	11 (9.6%)	0.26

*Pertinent laboratory findings on the day of admission to ICU*			
White blood cells × (10^9^/L), mean ± SD	11.9 ± 6.2	10.0 ± 5.5	0.05
Lymphocyte count × (10^9^/L), mean ± SD	0.8 (0.5, 1.3)	0.9 (0.6, 1.2)	0.93
Neutrophil count × (10^9^/L), mean ± SD	8.8 ± 4.2	7.6 ± 3.8	0.20
Hemoglobin (g/L), median (Q1, Q3)	122 (110, 135)	129 (119, 141)	0.04
Platelet × (10^9^/L), mean ± SD	275.2 ± 130.0	249.5 ± 93.3	0.18
Creatinine (*μ*mol/L), median (Q1, Q3)	105 (73, 162)	93 (72, 138)	0.19
Lactate (mmol/L), median (Q1, Q3)	1.85 (1.35, 2.66)	1.72 (1.15, 2.53)	0.28
Lactate dehydrogenase (U/L), median (Q1, Q3)	604 (476, 809)	643 (508, 824)	0.51
Ferritin (*μ*g/mL), median (Q1, Q3)	824 (463, 2067)	808 (505, 2097)	0.88
Fibrinogen (g/L), mean ± SD	5.6 ± 1.8	5.7 ± 1.6	0.74
D-dimer (*μ*g/mL), median (Q1, Q3)	1.8 (0.8, 4.0)	1.1 (0.7, 3.2)	0.21
C-reactive protein (mg/L), mean ± SD	160.4 ± 82.0	174.0 ± 111.8	0.77

*Medications*			
Corticosteroids, *N* (%)	48 (72.7%)	91 (79.1%)	0.33

BMI: body mass index, ICU: intensive care unit, Q1: first quartile, Q3: third quartile, and SD: standard deviation. Continuous variables with normal distribution are presented as mean with standard deviation and are compared using the independent *t*-test (significant at <0.05). Continuous variables with normal distribution are presented as median with the first and third quartile and are compared using Mann–Whitney test (significant at <0.05).

**Table 2:** Enteral nutrition parameters and signs of gastrointestinal intolerance in the study patients.

	Nonproned *N* = 66	Proned *N* = 115	*p* value
*Pre-NMBA day*			
Max EN volume in ml/hr	0 (0, 301)	0 (0, 13)	0.29
EN volume in ml/24 hr	0 (0, 712)	0 (0, 858)	0.47
Max GRV per check	28 (0, 86)	20 (0, 150)	0.52
GRV in ml/24 hr	65 (0, 170)	30 (681, 400)	0.77
No of vomiting episodes	0 (0, 0)	0 (0, 0)	1.0
No of bowel movements	0 (0, 0)	0 (0, 0)	0.99
Patients with diarrhea, *N* (%)	2 (3.0)	7 (6.1)	0.49

*NMB day 1*			
Max EN volume in ml/hr	30 (10, 52.5)	33(12, 60)	0.49
EN volume in ml/24 hr	320 (30, 720)	431 (50, 90)	0.49
Max GRV per check	0 (0, 70)	20 (0, 100)	0.16
GRV in ml/24 hr	0 (0, 145)	30 (0, 215)	0.16
No of vomiting episodes	0 (0, 0)	0 (0, 0)	1.0
No of bowel movements	0 (0, 1)	0 (0, 1)	0.96
Patients with diarrhea, *N* (%)	2 (3.0)	5 (4.3)	1.0

*NMB day 2*			
Max EN volume in ml/hr	40 (30, 60)	40 (30, 61)	0.79
EN volume in ml/24 hr	632 (355, 935)	635 (220, 898)	0.77
Max GRV per check	50 (20, 150)	75 (8, 173)	0.96
GRV in ml/24 hr	140 (30, 430)	120 (10, 400)	0.56
No of vomiting episodes	0 (0, 0)	0 (0, 0)	1.0
No of bowel movements	0 (0, 1)	0 (0, 0)	0.75
Patients with diarrhea, *N* (%)	1/63 (1.6)	4/109 (3.7)	0.65

*NMB day 3*			
Max EN volume in ml/hr	41 (30, 57)	40 (30, 60)	0.61
EN volume in ml/24 hr	646 (470, 1144)	713 (482, 1078)	0.81
Max GRV per check	70 (15, 175)	70 (20, 180)	0.95
GRV in ml/24 hr	170 (30, 390)	165 (33, 428)	0.83
No of vomiting episodes	0 (0, 0)	0 (0, 0)	0.41
No of bowel movements	0 (0, 0.2)	0 (0, 1)	0.52
Patients with diarrhea, *N* (%)	2/46 (4.3)	10 (81)	0.21

*NMB day 4*			
Max EN volume in ml/hr	44 (32, 56)	40 (30, 61)	0.63
EN volume in ml/24 hr	789 (561, 1101)	726 (528, 1116)	0.55
Max GRV per check	100 (28, 185)	80 (30, 180)	0.59
GRV in ml/24 hr	210 (75, 665)	200 (40, 470)	0.33
No of vomiting episodes	0 (0, 0)	0 (0, 0)	0.16
No of bowel movements	0 (0, 1)	0 (0, 1)	0.74
Patients with diarrhea, *N* (%)	5/39 (12.8)	7/75 (9.3)	0.54

*Post-NMBA day 1*			
Max EN volume in ml/hr	51 (34, 65)	50 (38, 69)	0.27
EN volume in ml/24 hr	934.5 (640, 1356)	980 (720, 1440)	0.41
Max GRV per check	80 (0, 150)	50 (0, 173)	1.0
GRV in ml/24 hr	250 (85, 395)	165 (60, 504)	0.95
Vomiting episodes	0 (0, 0)	0 (0, 0)	0.45
No of bowel movements	0 (0, 0.5)	0 (0, 1)	0.18
Patients with diarrhea, *N* (%)	5/62 (8.1)	11/110 (10.0)	0.68

*Post-NMBA day 2*			
Max EN volume in ml/hr	56 (35, 68)	50 (38, 68)	0.89
EN volume in ml/24 hr	1008 (702, 1432)	897 (706, 1424)	0.46
Max GRV per check	203.78 (10, 200)	50 (20, 160)	0.32
GRV in ml/24 hr	235 (10, 580)	118 (28, 347)	0.21
No of vomiting episodes	0 (0, 0)	0 (0, 0)	1.0
No of bowel movements	0 (0, 2)	0 (0, 2)	0.85
Patients with diarrhea, *N* (%)	7/56 (12.5)	13/92 (14.1)	0.78

EN: enteral nutrition, GRV: gastric residual volume, NMBA: neuromuscular blocking agent, Q1: first quartile, and Q3: third quartile. Continuous variables were presented as median with interquartile range (not normally distributed). Chi square test/Fisher's exact test was done for categorical variables (significant at <0.05). Mann–Whitney test was performed for all continuous variables (significant at <0.05).

**Table 3:** Outcomes of the study patients.

	Nonproned *N* = 66	Proned *N* = 115	*p* value
ICU-acquired pneumonia, *N* (%)	22 (37.9)	36 (31.3)	0.78
Tracheostomy, *N* (%)	14 (37.1)	22 (63.9)	0.74
Duration of MV, median (Q1, Q3)	13.0 (7.0, 20.0)	13.5 (8.0, 19.0)	0.87
ICU length of stay, median (Q1, Q3)	15.0 (8.5, 23.5)	17.00 (10.0, 24.0)	0.28
Hospital length of stay, median (Q1, Q3)	13.3 (22.0, 33.0)	22.0 (16.0, 34.0)	0.57
ICU death, *N* (%)	40 (60.6)	64 (55.6)	0.40
Hospital death, *N* (%)	43 (65.2)	68 (59.1)	0.32

ICU: intensive care unit, MV: mechanical ventilation, Q1: first quartile, and Q3: third quartile. Chi square test/Fisher's exact test was done for categorical variables (significant at <0.05). Mann–Whitney test was performed for all continuous variables (significant at <0.05).

## Data Availability

The data presented in this study are available upon request from the corresponding author. The data are not publicly available due to institutional policies of maintaining the confidentiality of patient data.
